# How do cardiovascular diseases harm labor force participation? Evidence of nationally representative survey data from Japan, a super-aged society

**DOI:** 10.1371/journal.pone.0219149

**Published:** 2019-07-05

**Authors:** Rong Fu, Haruko Noguchi, Shuhei Kaneko, Akira Kawamura, Cheolmin Kang, Hideto Takahashi, Nanako Tamiya

**Affiliations:** 1 Waseda University, Faculty of Political Science and Economics, Tokyo, Japan; 2 National Institute of Public Health, Saitama, Japan; 3 Health Services Research & Development Center, University of Tsukuba, Tsukuba, Japan; 4 Department of Health Services Research, Faculty of Medicine, University of Tsukuba, Tsukuba, Japan; University of South Australia, AUSTRALIA

## Abstract

**Objective:**

To evaluate how cardiovascular diseases harm labor force participation (LFP) among the Japanese population and verify the validity of plasma biomarkers as instrumental variables of cardiovascular diseases after adjusting for a broad set of confounders including dietary intake.

**Design:**

Using nationally representative repeated cross-sectional surveys in Japan, the Comprehensive Survey of Living Conditions and National Health and Nutrition Survey, with plasma biomarkers as instrumental variables for quasi-randomization.

**Setting:**

Onset of cardiovascular diseases in those receiving regular treatment for hypertension, intracerebral hemorrhage, intracerebral infarction, angina pectoris, myocardial infarction, or other types of cardiovascular diseases.

**Participants:**

A total of 65,615 persons aged ≥ 20 years (35,037 women and 30,578 men) who completed a survey conducted every three years from 1995 through 2013.

**Main outcome measures:**

Respondent employment and weekly working hours during each survey year.

**Results:**

Cardiovascular diseases significantly and remarkably reduced the probability of working by 15.4% (95% CI: -30.6% to -0.2%). The reduction in working probability was detected for women only. Respondents aged ≥ 40 years were less likely to work once diagnosed and the reduction was enlarged for those aged ≥ 65 years, while those aged < 40 years appeared to be unaffected. Probability of engaging in manual labor significantly decreased once diagnosed; however, no impact was found for cognitive occupations. Among employed respondents, the adverse effects of cardiovascular diseases decreased working hours by five hours per week. Validity of the biomarker instrumental variables was generally verified.

**Conclusions:**

A vicious circle is suggested between LFP and unfavorable health. However, the effects vary across age, sex, and occupation type, even after adjusting for causal effects, which could cause a downward bias in LFP impact.

**Attributes:**

cardiovascular disease, labor force participation, instrumental variable method as quasi-randomization, plasma biomarker, Comprehensive Survey of Living Conditions, National Health and Nutrition Survey.

## Introduction

Cardiovascular disease is one of the major causes of death and disability in both developed and developing countries [1,2]; according to the World Health Organization, the number of deaths from cardiovascular diseases in 2016 was 17.9 million, comprising over 31% of all global deaths [3]. In Japan, nationwide prevention and control of cardiovascular risks has successfully reduced related deaths from 40% of all deaths in 1980 to 25% in 2011; nevertheless, cardiovascular diseases impose a significant burden of physical and mental disability and pain on the population [4].

Cardiovascular diseases also place a substantial economic burden on society, in both increasing medical costs (i.e., direct costs), and productivity losses in the labor market (i.e., indirect costs) [5–8]. Most studies provide evidence of direct costs, while only few have examined indirect costs [9,10]—probably because the dual relationship between diseases and labor force participation (LFP) makes causality difficult to infer. For instance, work stress and long working hours significantly drive up cardiovascular risks [11]. Without adjusting for these confounding variables, it seems as if the onset of cardiovascular disease may promote LFP. Furthermore, using observational data, the causal inference could be ambiguous because of unobserved socioeconomic factors, such as dietary patterns, which would be associated with both cardiovascular risks and LFP [12]. Considering the global prevalence of cardiovascular diseases and the limited evidence on its indirect costs, it is of great interest to examine the causal effect of cardiovascular diseases on LFP.

To overcome the challenges in casual inference, an approach widely used in the literature is that of instrumental variables (IVs) [13]. This incorporates a group of third variables being firmly correlated to the risk of exposure but not to the outcome of interest. The causal inference can then be made because the only path by which the IVs could affect the outcome is through their correlations with the exposure. With the expansion of knowledge in biomedical science, an increasing number of studies in epidemiology, economics, and social science have been utilizing genetic markers as IVs [14–17]. Specifically, epidemiologists refer to the genetic-IV method as “Mendelian Randomization” since an individual’s genotype is randomly assigned at conception. Under appropriate assumptions, genotype could be exploited to show the causal relationship between health and socioeconomic outcomes because the observed impact of genotype on the outcome is possibly attributable to the correlation between the genotype and health [17,18].

Although genetic markers are plausible IVs, it is ethically difficult to access biomedical datasets, such as biobanks, that contain genotypic information, and the sample sizes of available datasets appear to be limited [19]. As an alternative, in this study, we utilized plasma biomarkers (PBs), such as plasma triglyceride, as the IVs for cardiovascular diseases. Compared to genotype, PBs are much more widely recorded in biomedical datasets, and a handful of preceding studies have incorporated PBs to the investigations. Some verified how socioeconomic status (e.g., education, income, and dietary intake) would contribute to PBs gradient [20,21]. Others showed how risky PB readings would affect socioeconomic status such as employment [22,23]. However, they have either implicitly equated PBs with diseases or regarded PBs as *ad hoc* risk factors of the diseases without probing deeper to show the real impact of PBs on diseases. Using PBs as IVs (PB-IVs), we can build on the literature, by not only revealing the LFP effects of cardiovascular diseases, but also simultaneously evaluating the extent that PBs are active as risk factors.

However, there are issues in using PB-IVs; among which, the major concern is about exogeneity. Unlike genotypes, PBs may be associated with socioeconomic confounders (e.g., dietary pattern and smoking) that are correlated with both health and LFP [24,25]. If the confounders are unobservable, incorporating PB-IVs would yield inconsistent estimates. To overcome the challenge, therefore, we particularly controlled for daily dietary intake, which is notably associated with the biomarker readings as well as LFP [26–29]. We verified the validity of PB-IVs after adjusting for related confounders, which could contribute to the literature by providing a wider range of possibilities to utilize PBs as IVs.

Most importantly, our findings build on the literature with evidence of the extent that cardiovascular diseases reduce LFP in Japan. Although several studies have found adverse effects of obesity or diabetes on LFP in reductions in probability of working and wages [30–33], they utilized datasets from Western countries such as the United States, European countries, and Australia [34–36]. Our findings from the Japanese population would therefore be beneficial in constructing a global understanding of the productivity loss produced by cardiovascular diseases.

## Estimation strategy

We applied the instrument variable method to investigate the effect of cardiovascular diseases on LFP. The estimation can be shown in a simple formula as below,

$$Y_{i}=\alpha_{0}+\alpha_{1}CD_{i}+\alpha_{2}X_{i}^{'}+\alpha_{t}T^{'}+\alpha_{p}P^{'}+\alpha_{tp}TP^{'}+u_{i},$$ (1)

where *Y*_*i*_ stands for the performance in the labor market for respondent *i*; *α*_0_ is the intercept; *CD*_*i*_ represents a dichotomous variable for the cardiovascular disease status, and *α*_1_ is thus the coefficient of interest; ***X***_***i***_ is a set of confounding covariates; ***T*** and ***P*** is a set of year and prefecture fixed effects, respectively; ***TP*′** is a set of interactions of the year and prefecture fixed effects; *u*_*i*_ represents the unobserved factors that may also associate with *Y*_*i*_. For each of the variables above, details are shown in Section 3.

The challenge we encounter when estimating Eq (1) is that *CD*_*i*_ may be associated with *u*_*i*_, that is, cov(*CD*_*i*_, *u*_*i*_) ≠ 0, and thus a naive ordinary least squares (OLS) estimation yields an inconsistent estimate of α_1_. We sought to tackle the issue by applying two-stage least squares (2SLS) regression, where the first-stage regression is,

$${CD}_{i}=\beta_{0}+\beta_{1}PB_{i}^{'}+\beta_{2}X_{i}^{'}+\beta_{t}T'+\beta_{p}P'+\beta_{tp}TP'+\nu_{i},$$ (2)

where *PB*_*i*_ is a vector of individual *i*’s PBs that are expected to be *valid* IVs, and *v*_i_ is the error term. To ensure the validity of the IVs, we assumed that the PB-IVs satisfy two restrictions: *relevance* (i.e., being correlated with *CDi* or **β**_**1**_ ≠ **0**); and *exclusion* (i.e., being uncorrelated with the error term in Eq (1) or cov(***PB***_***i***_, *u*_*i*_) = 0). Valid PB-IVs allow us to purify the effect of cardiovascular diseases on LFP because the PB-IVs do not interact with LFP other than through their effects on cardiovascular risks and observed associations between PB-IVs and LFP can be used consistently to infer causality.

The relevance assumption appears to be naturally satisfied since the PBs incorporated in this study are commonly found to be good indicators of cardiovascular risks. To improve the exclusion restriction, which may be violated by the potential correlation between PBs and unobserved factors, we explicitly included information about daily dietary intake in the covariates set ***X***_***i***_. Practically, valid PB-IVs allow us to estimate *α*_1_ consistently by substituting the predicted value of *CD*_*i*_ from Eq (2) into Eq (1). All estimations were conducted using user-written command–ivreg2– in Stata 15.1.

## Data and measurements

### Design and participants

We used data from the Comprehensive Survey of Living Conditions (CSLC) from 1995 to 2013, which is a nationally representative, repeated, cross-sectional survey of the non-institutionalized population in Japan. In addition, we linked the CSLC to the concurrent National Health and Nutrition Survey (NHNS), which was conducted with a randomly extracted subsample of the CSLC [37]. This research was conducted with permission from the Ethics Review Committees of Waseda University (approval no. 729–420).

The CSLC has been conducted once every three years in June as of 1986 by the Ministry of Health, Labour and Welfare (MHLW). Applying a two-stage random sampling procedure, it contains four questionnaires assessing household, health, income/saving, and long-term care. The questionnaire on household and health covers all respondents, comprising 600,000–800,000 people from approximately 300,000 households randomly selected in each survey year. The questionnaire on income/saving and long-term care complementarily covers a part of the full sample of respondents, including around 100,000 and 6,000 people, respectively. The NHNS—conducted in November of the survey year—also covers a part of the full CSLC respondents, comprising 8,000–10,000 people. For simplicity, we hereinafter denote the NHNS as a *nutrition* questionnaire of the CSLC.

In this study, we analyzed data from the questionnaire on household, health, and nutrition for the estimations. We excluded income/saving data because few respondents therein were recorded in the nutrition questionnaire as well. We did not use the long-term care questionnaire because the respondents therein are care needy and therefore highly likely to be economically inactive. We further excluded data from years before 1995, during which information in the nutrition questionnaire was aggregated at the household level rather than reported individually. Since the blood test in the nutrition questionnaire is conducted only with respondents aged ≥ 20 years, we removed those aged < 20 years. We eventually constructed a sample of 65,615 respondents (35,037 women and 30,578 men) over the seven survey years.

We obtained official permission to use the CSLC and NHNS from the MHLW (Tohatsu-0507-3 as of May 7, 2018) based on Article 32 of the Statistics Act. Ethical reviews of these data were not required, based on the Ethical Guidelines for Epidemiological Research of the Japanese government [38].

### Measurements

#### LFP

We used two variables to assess LFP: (1) “working” (1 = yes, 0 = otherwise) and (2) “weekly working hours;” that is, working hours in the week prior to the survey date. Measurement (1) refers to the extensive margin of LFP; that is, the probability of working, and (2) indicates the intensive margin; that is, the intensity of work to be supplied if working [39]. Both are positive indicators of LFP and thus were expected to be negatively associated with cardiovascular diseases.

#### Cardiovascular disease

We defined a dichotomous variable—cardiovascular disease—which took a value of one if a respondent visited a medical institution regularly for treatment for at least one of the following diseases: hypertension, intracerebral hemorrhage or intracerebral infarction, angina pectoris or myocardial infarction, or another type of cardiovascular disease. The variable took a value of zero if the respondent did not visit any medical institutions and was thus not diagnosed; that is, we excluded respondents reporting other diagnoses.

#### Biomarkers

We constructed four PB-IVs as follows. The first IV was dichotomous, taking a value of one if the level of plasma triglyceride (TG) was higher than 150 milligrams per deciliter (mg/dL), and otherwise a value of zero. TG is a significant risk factor of cardiovascular diseases, and a TG level below the threshold is regarded as normal; that is, not at risk [40]. Accordingly, this IV was expected to be a positive indicator of cardiovascular risks. The second IV was a ratio of the total cholesterol level (TC) to the level of high-density lipoprotein cholesterol (HDL-C). An increase in TC is considered to drive up the risk of cardiovascular diseases, while HDL-C is found to mitigate against the related risks [41]. The ratio, therefore, was expected to be positively associated with the cardiovascular risks [42]. The third IV was a dichotomous indicator of hypertension, taking a value of one if a respondent had a systolic blood pressure (SBP) reading above 140 millimeter of mercury (mmHg), or a diastolic blood pressure (DBP) reading above 90 mmHg, or both; otherwise, it took a value of zero. SBP and DBP above the thresholds are commonly used to diagnose hypertension [43]; thus the IV was expected to be positively associated with cardiovascular risks. The last IV was also dichotomous, taking a value of one if plasma glucose (GLU) level was higher than 110 mg/dL, and otherwise a value of zero. A threshold-above GLU significantly raises risks of type 2 diabetes, which is closely associated with cardiovascular diseases [44]. Accordingly, the IV was also a positive indicator of cardiovascular risks. The epidemiology literature suggests that PB-IVs are good indicators of cardiovascular diseases, that is, the relevance assumption is satisfied [40–44].

Nonetheless, we were concerned about the exclusion restriction since the PBs may interact with other factors. One significant factor is the postprandial hour of a blood test, since the readings of biomarkers, those of TG and GLU, fluctuate notably during the hours after a meal [45,46]. We thus controlled for the postprandial phase—during which the blood tests are conducted—where a blood test was categorized into the following eight postprandial bands (1 = yes, 0 = no): at about 30 minutes; at about one, two, three, or four hours; around five to six hours; around seven to eight hours; or more than eight hours. As discussed, dietary pattern is also a factor significantly associated with PBs [26–29]. We thus included information about dietary intake to improve the validity of the IVs.

#### Daily dietary intake

We controlled for 24 indicators of respondents’ daily dietary intake, which reflect their dietary pattern and may interact simultaneously with PBs and LFP. In addition to the overall energy intake (kilogram calorie, kcal), the indicators were classified into seven categories: (1) water (gram, g); (2) carbohydrate (g); (3) protein, including animal protein (g) and vegetable protein (g); (4) lipid/fat, including animal lipid (g), vegetable lipid (g), saturated fat (g), monounsaturated fat (g), and polyunsaturated fat (g); (5) minerals, including ash (g), sodium (milligram, mg), potassium (mg), calcium (mg), magnesium (mg), phosphorus (mg), and iron (mg); (6) vitamins, including vitamin D (microgram, μg), vitamin E (mg), vitamin B1 (mg), vitamin B2 (mg), niacin (mg), and vitamin C (mg); and (7) total dietary fiber (g).

#### Socioeconomic and health background

We stratified other socioeconomic factors into a group standing for individual status and another for household status, respectively. Regarding individual status, we controlled for sex (1 = men, 0 = women); age in years; being the household head (1 = yes, 0 = no); being the main caregiver to a co-resident family member who needs care (1 = yes, 0 = no); each of the following marital status categories (1 = yes, 0 = no): married, single, widowed, and divorced; and each of the following categories of pension enrollment (1 = yes, 0 = no): category I enrollee (i.e., self-employed people), category II enrollee (i.e., salaried workers or government employees), category III enrollee (i.e., dependents of a category II enrollee), and not enrolled in the pension system.

Regarding household socioeconomic status, we controlled for monthly household expenditure (log-scaled) in each of the following household structure categories (1 = yes, 0 = no): single family, couple, couple with children, single parent with children, three generations, and other types; and in each of the following types of residence (1 = yes, 0 = no): owned house, private rental house, issued house, public rental house, and other types, and living spaces in square meters (m^2^).

We also adjusted for health status and health behavior as potential confounders. Regarding health, we controlled for stressful feelings in daily life (1 = yes, 0 = no); self-reported restricted daily life because of poor health (1 = yes, 0 = no); having subjective symptoms (1 = yes, 0 = no); body mass index (BMI); and the following levels of self-rated health status (1 = yes, 0 = no): very good, good, fair, bad, and very bad. Regarding health behavior, we controlled for smoking (1 = yes, 0 = no), daily step counts, having regular exercise (1 = yes, 0 = no), and having regular health check-ups (1 = yes, 0 = no).

## Results

### Basic statistics

Table 1 summarizes basic statistics for LFP, cardiovascular diseases, the PB-IVs, postprandial phase, dietary intake, socioeconomic background, and health/health behavior over the seven survey years. Proportion of working respondents in the full sample was 59%, where those with cardiovascular diseases were less likely to be working than healthy respondents (40% versus 66%). A similar gap can be found for weekly working hours: compared to healthy respondents, cardiovascular events tended to decrease weekly working hours by 4.3 hours.

**Table 1:** Basic statistics by cardiovascular event^a^^,^ ^b^^,^ ^c^.

	Total			Cardiovascular event			No cardiovascular event			T-test
	n	Mean	SD	n	Mean	SD	n	Mean	SD	
**Labor force participation**										
Working	59,067	0.59	(0.49)	5,266	0.40	(0.49)	34,853	0.66	(0.47)	***
Weekly working hours	15,712	40.05	(16.92)	1,495	36.88	(18.39)	9,920	41.14	(16.41)	***
**Cardiovascular diseases**	40,778	0.13	(0.34)							
**Biomarkers**										
TG > 150 mg/dL	31,799	0.30	(0.46)	3,060	0.33	(0.47)	16,542	0.28	(0.45)	***
TC/HDL-C	31,799	3.60	(1.15)	3,060	3.61	(1.06)	16,542	3.56	(1.15)	***
SBP > 140 mmHg or DBP > 90 mmHg	38,033	0.34	(0.47)	3,417	0.50	(0.50)	19,675	0.27	(0.45)	***
GLU > 110 mg/dL	31,649	0.24	(0.43)	3,046	0.36	(0.48)	16,478	0.19	(0.39)	***
Postprandial phase (ref: about 30 minutes)										
About 1 hour	31,276	0.07	(0.25)	3,020	0.06	(0.24)	16,298	0.07	(0.25)	***
About 2 hours	31,276	0.11	(0.31)	3,020	0.13	(0.33)	16,298	0.10	(0.30)	***
About 3 hours	31,276	0.16	(0.37)	3,020	0.20	(0.40)	16,298	0.14	(0.35)	***
About 4 hours	31,276	0.20	(0.40)	3,020	0.24	(0.43)	16,298	0.18	(0.39)	***
5–6 hours	31,276	0.28	(0.45)	3,020	0.23	(0.42)	16,298	0.30	(0.46)	***
7–8 hours	31,276	0.08	(0.27)	3,020	0.05	(0.21)	16,298	0.09	(0.29)	***
> 8 hours	31,276	0.03	(0.17)	3,020	0.04	(0.18)	16,298	0.03	(0.17)	***
**Daily dietary intake**										
Energy (kcal)	62,589	1968.06	(600.40)	5,098	1887.81	(550.16)	33,822	2013.99	(613.79)	***
Water (g)	62,590	1385.80	(632.60)	5,098	1600.84	(646.29)	33,823	1353.24	(625.73)	***
Carbohydrate (g)	62,590	273.20	(87.30)	5,098	268.92	(81.00)	33,823	275.87	(88.88)	***
Animal protein (g)	62,427	40.56	(20.40)	5,082	38.32	(19.28)	33,743	41.60	(20.71)	***
Vegetable protein (g)	62,590	35.03	(12.00)	5,098	34.21	(11.27)	33,823	35.35	(12.12)	***
Animal lipid (g)	62,427	27.69	(16.50)	5,082	25.05	(14.93)	33,743	29.07	(17.12)	***
Vegetable lipid (g)	62,588	28.09	(15.60)	5,098	25.01	(14.07)	33,821	29.35	(15.97)	***
Saturated fat (g)	62,587	15.30	(8.20)	5,098	13.27	(6.95)	33,820	16.16	(8.48)	***
Monounsaturated fat (g)	62,587	18.84	(9.60)	5,098	16.52	(8.49)	33,820	19.91	(9.84)	***
Polyunsaturated fat (g)	62,587	13.28	(6.40)	5,098	11.78	(5.73)	33,820	13.82	(6.52)	***
Ash (g)	62,589	20.11	(7.90)	5,098	19.37	(7.04)	33,822	20.24	(7.96)	***
Sodium (mg)	62,590	4724.56	(2140.10)	5,098	4479.02	(1940.69)	33,823	4782.75	(2176.78)	***
Potassium (mg)	62,590	2580.91	(1062.70)	5,098	2607.02	(1014.62)	33,823	2561.00	(1057.33)	***
Calcium (mg)	62,590	532.63	(280.80)	5,098	563.77	(280.04)	33,823	520.70	(275.87)	***
Magnesium (mg)	62,586	266.96	(110.60)	5,098	270.83	(99.78)	33,820	266.04	(112.46)	***
Phosphorus (mg)	62,589	1086.88	(388.40)	5,098	1052.83	(362.94)	33,822	1098.82	(391.64)	***
Iron (mg)	62,587	9.57	(4.90)	5,098	9.01	(4.07)	33,821	9.67	(5.16)	**
Vitamin D (μg)	61,154	48.86	(121.80)	5,048	25.69	(79.06)	33,006	52.94	(128.90)	***
Vitamin E (mg)	62,587	9.01	(13.10)	5,097	9.00	(17.76)	33,821	8.93	(10.82)	*
Vitamin B1 (mg)	62,588	1.25	(4.00)	5,098	1.44	(5.32)	33,821	1.21	(3.52)	**
Vitamin B2 (mg)	62,590	1.40	(2.80)	5,098	1.41	(1.80)	33,823	1.39	(2.40)	**
Niacin (mg)	62,590	16.40	(8.00)	5,098	15.86	(7.80)	33,823	16.72	(8.06)	***
Vitamin C (mg)	62,560	121.25	(124.60)	5,093	135.05	(122.87)	33,807	115.36	(120.97)	***
Total dietary fiber (g)	62,583	15.15	(7.00)	5,098	16.44	(7.22)	33,820	14.75	(6.77)	***
**Socioeconomic status: individual**										
Men	65,615	0.47	(0.50)	5,414	0.49	(0.50)	35,364	0.49	(0.50)	
Age in years	65,605	50.34	(18.67)	5,411	66.64	(13.61)	35,361	44.66	(16.81)	***
Being household head	60,049	0.43	(0.50)	5,414	0.57	(0.49)	35,364	0.39	(0.49)	***
Being main caregiver	59,927	0.03	(0.16)	5,410	0.08	(0.27)	35,267	0.01	(0.08)	***
Marital status (ref: married)										
Single	60,049	0.20	(0.40)	5,414	0.05	(0.22)	35,364	0.26	(0.44)	***
Widowed	60,049	0.08	(0.26)	5,414	0.17	(0.38)	35,364	0.04	(0.19)	***
Divorced	60,049	0.03	(0.17)	5,414	0.04	(0.19)	35,364	0.03	(0.16)	**
Pension enrollment (ref: category I enrollee)										
Category II enrollee	48,649	0.19	(0.39)	4,668	0.08	(0.28)	29,759	0.21	(0.41)	***
Category III enrollee	48,649	0.39	(0.49)	4,668	0.20	(0.40)	29,759	0.45	(0.50)	***
Not enrolled	48,649	0.10	(0.29)	4,668	0.03	(0.18)	29,759	0.11	(0.31)	***
**Socioeconomic status: household**										
Household expenditures per month (10 thousand yen)	55,748	30.34	(35.52)	5,092	26.48	(29.86)	32,900	30.89	(35.22)	***
Household structure (ref: single family)										
Couple	60,049	0.20	(0.40)	5,414	0.36	(0.48)	35,364	0.15	(0.36)	***
Couple with child(ren)	60,049	0.40	(0.49)	5,414	0.22	(0.41)	35,364	0.47	(0.50)	***
Single parent with child(ren)	60,049	0.04	(0.20)	5,414	0.04	(0.20)	35,364	0.04	(0.21)	
Three generations	60,049	0.20	(0.40)	5,414	0.16	(0.37)	35,364	0.21	(0.41)	***
Others	60,049	0.07	(0.26)	5,414	0.10	(0.30)	35,364	0.06	(0.24)	***
Residence (ref: owned house)										
Private rental house	60,049	0.11	(0.31)	5,414	0.07	(0.25)	35,364	0.13	(0.33)	***
Issued house	60,049	0.03	(0.16)	5,414	0.01	(0.08)	35,364	0.03	(0.18)	***
Public rental house	60,049	0.05	(0.21)	5,414	0.05	(0.22)	35,364	0.05	(0.21)	
Others	60,049	0.02	(0.14)	5,414	0.02	(0.14)	35,364	0.02	(0.14)	
Living space (m^2^)	58,046	95.93	(85.86)	5,244	109.73	(77.37)	34,215	92.41	(69.28)	***
**Health and health behavior**										
Feeling stressed in daily life	55,298	0.48	(0.50)	4,969	0.54	(0.50)	33,789	0.44	(0.50)	***
Having restricted daily life because of poor health	54,983	0.12	(0.32)	4,842	0.29	(0.46)	33,836	0.03	(0.18)	***
Having subjective symptoms	58,780	0.35	(0.48)	5,414	0.59	(0.49)	35,364	0.19	(0.40)	***
BMI	54,473	22.78	(3.43)	4,611	23.75	(3.60)	29,240	22.54	(3.34)	***
Self-rated health status (ref: very good)										
Good	55,264	0.17	(0.38)	4,949	0.13	(0.33)	33,805	0.19	(0.39)	***
Fair	55,264	0.46	(0.50)	4,949	0.51	(0.50)	33,805	0.45	(0.50)	***
Bad	55,264	0.12	(0.32)	4,949	0.25	(0.43)	33,805	0.05	(0.22)	***
Very bad	55,264	0.01	(0.12)	4,949	0.04	(0.20)	33,805	0.00	(0.06)	***
Smoking	35,118	0.27	(0.44)	4,314	0.18	(0.38)	20,077	0.31	(0.46)	***
Step counts per day	57,490	7145.24	(4288.06)	4,719	5831.81	(4007.76)	31,116	7640.61	(4248.26)	***
Regular exercise	43,026	0.23	(0.42)	2,963	0.31	(0.46)	23,463	0.20	(0.40)	***
Regular health check	53,826	0.39	(0.49)	5,163	0.40	(0.49)	31,822	0.41	(0.49)	***

^a^ HDL, high density lipoprotein

^b^ SD, standard deviation.

^c^ Inference: *p < .1, **p < .05, ***p < .01.

As expected, the PB-IVs predicted risk of cardiovascular diseases well. For instance, 33% of those with cardiovascular diseases had TG over 150 mg/dL, which was higher by about 5% than healthy respondents. The rest of Table 1 shows the differences in individual socioeconomic and health status by the onset of cardiovascular diseases.

### Effects on probability of working

Table 2 illustrates the LFP impact of cardiovascular diseases in reduction of the probability of working. In addition to the 2SLS, we reported results from OLS as a reference. For 2SLS, we reported coefficients on the PB-IVs in the first-stage regression, together with statistics for the weak-identification and overidentification tests to verify the validity of the IVs.

**Table 2:** Influences of cardiovascular diseases on the probability of working^a^^,^ ^b^^,^ ^c^.

	OLS (n = 21,163)		2SLS (n = 13,584)	
Cardiovascular disease	-0.025	***	-0.154	**
	(0.01)		(0.07)	
	[-0.043, -0.006]		[-0.306, -0.002]	
**First stage**				
TG > 150 mg/dL			0.015	*
			(0.01)	
			[-0.009, 0.039]	
TC/HDL-C			0.020	***
			(0.00)	
			[0.015, 0.026]	
SBP > 140 mmHg or DBP > 90 mmHg			0.124	***
			(0.01)	
			[0.095, 0.153]	
GLU > 110 mg/dL			0.128	***
			(0.01)	
			[0.098, 0.158]	
**Covariates**				
Socioeconomic status	Yes		Yes	
Health and health behavior	Yes		Yes	
Postprandial phase	Yes		Yes	
Daily dietary intake	Yes		Yes	
Year fixed effect	Yes		Yes	
Prefecture fixed effect	Yes		Yes	
**Weak identification**				
Cragg-Donald Wald F			44.586	
Kleibergen-Paap rk Wald F			37.882	
**Overidentification**				
Hansen J statistic			5.885	
p-value			.164	
**F statistics**	59.18		42.39	

^a^ Robust standard errors for individual heteroscedasticity in parentheses.

^b^ 95% confidence intervals in brackets.

^c^ Inference: *p < .1, **p < .05, ***p < .01.

At first glance, the absolute value of the OLS estimates appeared much smaller than what was derived by 2SLS, indicating a downward bias of the LFP impact because of the correlation between cardiovascular diseases and unobserved factors. Concentrating on 2SLS estimates then, we found that having cardiovascular diseases significantly reduced the probability of working—the extensive margin of labor supply—by around 15.4% (95% CI: -30.6% to -0.2%). It is worthwhile mentioning that 2SLS treated the binary outcome “working” as continuous. To test the robustness of the 2SLS results, we applied a bivariate probit model where the second-stage regression was modelled in a probit fashion (see Table A in S1 Appendix). After adjusting for the binary outcome, the marginal effect calculated from the bivariate probit estimation was close to the main results: a reduction in working probability by 12.6% (CI: -22.6% to -2.6%). As detailed below, we then focused on the findings from 2SLS regarding the validity of the IVs.

The relevance assumption for 2SLS was verified because the statistics of weak identification were commonly above the rule-of-thumb level of 10 [47]. This was also supported by the coefficients on PB-IVs in the first stage regression. All the PB-IVs significantly increased the risks of cardiovascular diseases; having CLU over 110 mg/dL raised the risk by over 12.8% (95% CI: 9.8% to 15.8%). Furthermore, we found that 2SLS failed to reject the null hypothesis of Hansen J test, suggesting that the PB-IVs are uncorrelated with the second-stage error term.

Based on the fully constructed 2SLS, we also implemented the estimation separately by sex, age band, and occupation type to trace variations of the LFP impact (Table 3). We stratified the data into male and female respondents; into those aged < 40 years, aged 40–65 years, and aged > 65 years. In addition, we stratified the data based on occupation type. One type was denoted as cognitive, where the variable “working” takes a value of one if a respondent is working *and* has one of the following occupations: administrative or managerial workers, specialist professionals, clerical workers, sales workers, and service workers; otherwise, it took a value of zero (i.e., if a respondent was not working). Another occupation type was manual, where the variable takes a value of one if a working respondent has one of the following occupations: security workers, agriculture, forestry, and fishery workers, manufacturing process workers, transport and machine operation workers, construction and mining workers, carrying, and cleaning workers; otherwise, it takes a value of zero (i.e., if a respondent is not working). In short, a value of zero for “working” here is identical to the initial definition, while a value of one denotes working in either occupation type—unlike the initial definition where a value of one simply indicates working.

**Table 3:** Influences of cardiovascular diseases on probability of working: By sex, age, and occupation type^a^^,^ ^b^^,^ ^c^^,^ ^d^.

	Sex				Age					Occupation type			
	Men (n = 5,714)		Women (n = 7,870)		< 40- (n = 4,136)	40–65 (n = 7,172)		> 65 (n = 2,276)		Cognitive (n = 9,172)		Manual (n = 8,278)	
Cardiovascular disease	-0.092		-0.191	*	-0.093	-0.162	**	-0.214	*	-0.085		-0.199	**
	(0.13)		(0.09)		(0.45)	(0.07)		(0.13)		(0.12)		(0.09)	
	[-0.321, 0.137]		[-0.386, 0.004]		[-0.584, 0.398]	[-0.292, -0.032]		[-0.431, 0.003]		[-0.320, 0.151]		[-0.385, -0.012]	
**First stage**													
TG > 150 mg/dL	0.003		0.000		0.002	0.012	*	0.007		0.005		0.005	
	(0.01)		(0.01)		(0.01)	(0.01)		(0.02)		(0.01)		(0.01)	
	[-0.016, 0.022]		[-0.019, 0.019]		[-0.009, 0.013]	[-0.005, 0.029]		[-0.039, 0.053]		[-0.012, 0.022]		[-0.013, 0.023]	
TC/HDL-C	0.021	***	0.021	***	0.000	0.019	***	0.048	***	0.023	***	0.023	***
	(0.00)		(0.00)		(0.00)	(0.00)		(0.01)		(0.00)		(0.00)	
	[0.013, 0.029]		[0.013, 0.030]		[-0.004, 0.005]	[0.011, 0.026]		[0.027, 0.070]		[0.016, 0.030]		[0.016, 0.031]	
SBP > 140 mmHg or DBP > 90 mmHg	0.088	***	0.140	***	0.010	0.130	***	0.017		0.122	***	0.127	***
	(0.01)		(0.01)		(0.01)	(0.01)		(0.02)		(0.01)		(0.01)	
	[0.041, 0.128]		[0.080, 0.199]		[-0.003, 0.022]	[0.094, 0.166]		[-0.022, 0.055]		[0.074, 0.169]		[0.079, 0.174]	
GLU > 110 mg/dL	0.096	***	0.139	***	0.005	0.234	***	0.244	**	0.129	***	0.211	***
	(0.01)		(0.01)		(0.01)	(0.04)		(0.10)		(0.01)		(0.04)	
	[0.056, 0.135]		[0.081, 0.197]		[-0.008, 0.018]	[0.159, 0.308]		[0.044, 0.443]		[0.071, 0.188]		[0.139, 0.283]	
**Covariates**													
Socioeconomic status	Yes		Yes		Yes	Yes		Yes		Yes		Yes	
Health and health behavior	Yes		Yes		Yes	Yes		Yes		Yes		Yes	
Postprandial phase	Yes		Yes		Yes	Yes		Yes		Yes		Yes	
Daily dietary intake	Yes		Yes		Yes	Yes		Yes		Yes		Yes	
Year fixed effect	Yes		Yes		Yes	Yes		Yes		Yes		Yes	
Prefecture fixed effect	Yes		Yes		Yes	Yes		Yes		Yes		Yes	
**Weak identification**													
Cragg-Donald Wald F	19.308		21.209		13.371	17.586		15.352		18.141		19.534	
Kleibergen-Paap rk Wald F	18.838		16.505		10.833	15.202		13.332		15.233		17.525	
**Overidentification**													
Hansen J statistic	0.918		5.002		3.957	7.157		1.718		4.755		5.147	
p-value	1.012		.243		.526	0172		.860		.482		.177	
F Statistics	38.57		113.98		75.09	36.13		138.99		301.72		156.43	

^a^ Estimations are implemented using 2SLS.

^b^ Robust standard errors in parentheses.

^c^ 95% confidence intervals in brackets.

^d^ Inference: *p < .1, **p < .05, ***p < .01

Sex-specific results verified that women were more likely to quit the labor market than were men once diagnosed with cardiovascular diseases. Specifically, the reduction in working probability for women was 19.1% (95% CI: -38.6% to 0.4%), which was moderately significant; however, for men, it was non-significant and lower by 9.2% (95% CI: -32.1% to 13.7%). Estimations by age band showed variations as well. The working probability for respondents aged < 40 years was not significantly affected by cardiovascular diseases, while it was significantly reduced by 16.2% (95% CI: -29.2% to -3.2%) for those aged 40–65 years. The decline expanded further to 21.4% (95% CI: -43.1% to 0.3%) for those aged > 65 years; however, the significance became moderate. Results by occupation type showed that cardiovascular diseases reduced the probability of working in a manual occupation by 19.9% (95% CI: -38.5% to -1.2%). Probability of performing cognitive work, in contrast, was not significantly affected. For robustness check, we also applied bivariate probit model to each of the stratified samples (see Table B in S1 Appendix). The bivariate-probit estimates were close to the main results in the sign, the magnitude, and the significance. The validity of the IVs was commonly verified by the corresponding statistics.

### Effects on working hours

Concentrating on respondents who were working, we then examined whether and to what extent cardiovascular diseases would affect the intensive margin of labor supply (Table 4). Nevertheless, the OLS estimate experience downward bias owing to overlooking the association between cardiovascular risks and unobserved factors.

**Table 4:** Influences of cardiovascular diseases on weekly working hours^a^^,^ ^b^^,^ ^c^.

	OLS (n = 6,575)		2SLS (n = 2,383)	
Cardiovascular disease	-2.960	***	-4.964	*
	(0.67)		(2.46)	
	[-4.272, -1.647]		[-10.228, 0.300]	
**First stage**				
TG > 150 mg/dL			0.010	
			(0.02)	
			[-0.019, 0.039]	
TC/HDL-C			0.032	***
			(0.01)	
			[0.019, 0.045]	
SBP > 140 mmHg or DBP > 90 mmHg			0.055	***
			(0.01)	
			[0.026, 0.083]	
GLU > 110 mg/dL			0.071	**
			(0.02)	
			[0.015, 0.126]	
**Covariates**				
Socio-economics status	Yes		Yes	
Health behavior	Yes		Yes	
Postprandial phase	Yes		Yes	
Daily dietary intake	Yes		Yes	
Year fixed effect	Yes		Yes	
Prefecture fixed effect	Yes		Yes	
**Weak identification**				
Cragg-Donald Wald F			14.222	
Kleibergen-Paap rk Wald F			12.368	
**Overidentification**				
Hansen J statistic			3.784	
p-value			.163	
**F Statistics**	48.55		90.78	

^a^ Robust standard errors in parentheses.

^b^ 95% confidence intervals in brackets.

^c^ Inference: *p < .1, **p < .05, ***p < .01.

Turning then to the 2SLS estimates, we found a significant reduction in work supply by 4.96 hours (95% CI: -10.23 hours to 0.3 hours) per week for respondents diagnosed with cardiovascular diseases. Owing to the limited sample size (N = 2,383), we did not stratify the estimation further into specific groups to avoid problematic statistical inference.

## Discussion

### Principal findings

The negative impact of cardiovascular disease on LFP events has been documented by several studies in Western countries [34–36]. In the present study, we built on these studies by providing new evidence from Japan, a super-aged society. Incorporating PBs as IVs while controlling for a set of crucial confounders such as daily dietary intake, we found the following: (1) cardiovascular diseases significantly and remarkably reduced the probability of working by 15.4%; (2) the reduction in probability of working was detected for women only; (3) respondents aged ≥ 40 years were less likely to work once diagnosed with cardiovascular disease and the reduction was enlarged for those ≥ aged 65 years, while those aged < 40 years appeared to be unaffected; (4) the probability of being a manual worker was significantly reduced once diagnosed; however, there was no impact verified for cognitive occupations; and (5) among employed respondents, the adverse impact of cardiovascular diseases was found to be a 5-hour decrease in working hours per week.

### Strengths of this study, possible mechanisms, and implications

Finding (1) indicates that cardiovascular disease negatively affects LFP. Studies in Japan have continuously shown a negative association between diseases (both physical and mental) and probability of working [48]. In these studies, however, the mechanisms behind the relationship are not obvious. As discussed, it is possible that diseases reduce the likelihood of working, or job loss has negative effects on health, or both. One study in Japan followed unemployed people for two years and found an adverse mental health impact of prolonged bouts of unemployment [49]. Ours, in turn, demonstrates that the opposite is also true. Taken together, we notice a feedback loop between health shocks and LFP.

Findings (2) and (3) decompose the adverse LFP effects by sex and age groups, respectively. Studies in Western countries have found a stronger adverse effect on probability of working for men than women. The adverse effect was also found to be larger for middle-aged adults compared to their younger counterparts. Specifically, a study in Australia [35] found that cardiovascular diseases reduced the probability of working by 4%, but only for men aged 50–64 years. The probability of working for women and men aged < 50 years, on the other hand, was not sensitive to cardiovascular events.

Regarding the sex difference, we showed the complete opposite in Japan. Japanese men who experienced cardiovascular diseases report the same statistical likelihood of working compared to their healthy counterparts, which is probably a result of the strong labor force attachment among this cohort. In contrast, women in Japan, those who are of child-bearing age or those who are middle-aged, are weakly attached to the labor market, and thus appear to be highly sensitive to health shocks such as cardiovascular diseases [50]. Regarding the age-specific difference, our findings are in line with previous studies, showing an accelerating deterioration in working probability with age. This may also be attributable to the diminishing labor force attachment as people age. It is intuitive that young adults are much more closely attached to the labor force than those about to retire, and they would be the least willing to quit the labor market after cardiovascular diseases.

Finding (4) is new to the literature. Although studies have revealed a mild cognitive impairment associated with cardiovascular diseases [51], this impairment does not appear to directly force Japanese people to leave cognitive occupations. Indeed, cognitive loss due to cardiovascular diseases may be less of a concern compared to the significant physical impairments, such as inflammation, limited mobility, and disability [52], which are verified to lead to a remarkable decline in the probability of working in manual occupations.

Finding (5) indicates an intensity loss due to cardiovascular burden among the working population. The literature in Japan has concluded that there is a trivial reduction in working intensity after cardiovascular diseases. For instance, one study [53] reported a 0.1-hour loss per month from heart or circulatory diseases, which is negligible compared to the decline due to others; for example, a 12.4-hour loss for depression. Concerning potential causality, our findings reveal that the adverse impact of cardiovascular diseases on working intensity is much larger than is commonly expected.

### Limitations

This study had several limitations. First, we could not observe a potential quality loss of LFP because of data limitations. The literature has revealed a negative impact of health shocks on LFP quality, such as willingness to work and work satisfaction. In addition to the decline in working intensity examined in this study, a loss of LFP quality may further impair working performance among people who have cardiovascular diseases and choose to stay in the labor force. Owing to the data limitations, we could not observe potential wage discrimination against people exposed to cardiovascular diseases. We could neither rule out the possibility of measurement error except for the PBs, as our data were collected from self-reported and retrospective surveys. Furthermore, we could not conduct sex-specific analyses on the working-hour impact of the cardiovascular diseases owing to the restricted sample size. For the same reason, we could not derive statistically plausible inferences for LFP impacts of specific components of the cardiovascular diseases. Further investigations with more comprehensive and accurate measurements of labor market outcomes and socioeconomic backgrounds are necessary to elucidate the adverse LFP impact of cardiovascular diseases.

## Supporting information

S1 Appendix Tables A and B. (PDF)
