# Supplementary material for: How do cardiovascular diseases harm labor force participation? Evidence of nationally representative survey data from Japan, a super-aged society
Source: PLoS One. 2019 Jul 5;14(7):e0219149. doi: 10.1371/journal.pone.0219149 (PMC6611572; doi:10.1371/journal.pone.0219149)
Supplement: S1 Appendix — (PDF) [file pone.0219149.s001.pdf]

How Do Cardiovascular Diseases Harm Labor Force Participation? Evidence of Nationally Representative Survey Data from Japan, a Super-aged Society

Rong Fu, Haruko Noguchi, Shuhei Kaneko, Akira Kawamura, Cheolmin Kang, Hideto Takahashi, Nanako Tamiya

Online Appendix

Table A. Influences of cardiovascular diseases on probability of working (N = 21,163)

	OLS		2SLS		Bivariate probit	
Cardiovascular disease	-0.025	***	-0.154	**	-0.126	***
	[-0.043, -0.006]		[-0.306, -0.002]		[-0.226, -0.026]	
**First stage**						
TG > 150 mg/dL			0.015	*	0.012	
			[-0.009, 0.039]		[-0.015, 0.004]	
TC/HDL-C			0.020	***	0.019	***
			[0.015, 0.026]		[0.014, 0.025]	
SBP > 140 mmHg or DBP > 90 mmHg			0.124	***	0.162	***
			[0.095, 0.153]		[0.066, 0.257]	
GLU > 110 mg/dL			0.128	***	0.173	***
			[0.098, 0.158]		[0.068, 0.277]	

Notes: Marginal effects derived for bivariate probit regression. Specifically, marginal effects of plasma biomarkers on cardiovascular diagnoses are derived by using command -margins- with option -predict (pmarg2), which calculates the marginal success probability for the second stage.*Inference: *p < .1, **p < .05, ***p < .01.

Table B. Influences of cardiovascular diseases on working probability: by sex, age, and occupation type

	**Sex**		**Age (years)**			**Occupation type**	
	**Men**	**Women**	**< 40**	**40–65**	**> 65**	**Cognitive**	**Manual**
2SLS	-0.092	-0.191 *	-0.093	-0.162 **	-0.214 *	-0.085	-0.199 **
	[-0.321, 0.137]	[-0.386, 0.004]	[-0.584, 0.398]	[-0.292, -0.032]	[-0.431, 0.003]	[-0.320, 0.151]	[-0.385, -0.012]
Bivariate probit	-0.079	-0.146 **	-0.052	-0.117 **	-0.197	-0.082	-0.184 **
	[-0.289, 0.131]	[-0.294, 0.002]	[-0.417, 0.313]	[-0.193, -0.041]	[-0.483, 0.089]	[-0.298, 0.133]	[-0.339, -0.030]
Observations	5,714	7,870	4,136	7,172	2,276	9,172	8,278

Notes: *Inference: *p < .1, **p < .05, ***p < .01.
